# The Domain-Specific Neural Basis of Auditory Statistical Learning in 5–7-Year-Old Children

**DOI:** 10.1162/nol_a_00156

**Published:** 2024-10-28

**Authors:** Tengwen Fan, Will Decker, Julie Schneider

**Affiliations:** Department of Communications Sciences and Disorders, Louisiana State University, Baton Rouge, LA, USA; Department of Psychology, Georgia Tech University, Atlanta, GA, USA; School of Education and Information Studies, University of California, Los Angeles, Los Angeles, CA, USA

**Keywords:** development, fMRI, language, statistical learning

## Abstract

Statistical learning (SL) is the ability to rapidly track statistical regularities and learn patterns in the environment. Recent studies show that SL is constrained by domain-specific features, rather than being a uniform learning mechanism across domains and modalities. This domain-specificity has been reflected at the neural level, as SL occurs in regions primarily involved in processing of specific modalities or domains of input. However, our understanding of how SL is constrained by domain-specific features in the developing brain is severely lacking. The present study aims to identify the functional neural profiles of auditory SL of linguistic and nonlinguistic regularities among children. Thirty children between 5 and 7 years old completed an auditory fMRI SL task containing interwoven sequences of structured and random syllable/tone sequences. Using traditional group univariate analyses and a group-constrained subject-specific analysis, frontal and temporal cortices showed significant activation when processing structured versus random sequences across both linguistic and nonlinguistic domains. However, conjunction analyses failed to identify overlapping neural indices across domains. These findings are the first to compare brain regions supporting SL of linguistic and nonlinguistic regularities in the developing brain and indicate that auditory SL among developing children may be constrained by domain-specific features.

## INTRODUCTION

Statistical learning (SL) is the ability to extract and monitor regularities from the environment across time and is often considered a fundamental mechanism of learning (Sherman et al., 2020). Although SL is typically considered a domain-general learning mechanism (e.g., Abla et al., 2008; Kirkham et al., 2002; Saffran et al., 1996, 1999), recent research indicates that there are domain-specific mechanisms supporting SL at both the behavioral and neural levels (Conway, 2020; Frost et al., 2015). In behavioral studies, SL performance varies depending on stimuli type, across both domain (linguistic vs. nonlinguistic; e.g., Siegelman & Frost, 2015) and modality (e.g., visual vs. auditory; Emberson et al., 2019). In studies investigating the neural mechanisms of SL, different brain activation patterns are induced by these different types of stimuli (McNealy et al., 2006; Turk-Browne et al., 2009), even in the absence of clear behavioral differences (McNealy et al., 2006, 2010, 2011; Scott-Van Zeeland et al., 2010). However, evidence for the synergistic relationship between domain-general and domain-specific mechanisms at the neural level exists mostly in research with adults. Even among adults, most research has focused on a single domain (linguistic: McNealy et al., 2006, 2010; nonlinguistic: Abla & Okanoya, 2008) or modality (visual: Schapiro et al., 2014; Turk-Browne et al., 2009; auditory: McNealy et al., 2006, 2010). To date, only two studies have directly compared the neural basis of linguistic and nonlinguistic SL, identifying both domain-general and domain-specific neural mechanisms of auditory SL in adults (Schneider et al., 2024; Tremblay et al., 2013). The domain specificity of the neural basis of SL, however, remains largely unaddressed in the developing brain.

Early behavioral investigations of SL support a domain-general account, as SL has been observed across modalities (e.g., visual: Kirkham et al., 2002; auditory: Saffran et al., 1996), domains (e.g., linguistic: Saffran et al., 1996; nonlinguistic: Abla et al., 2008; Saffran et al., 1999), age groups (e.g., infant: Saffran et al., 1996; children: McNealy et al., 2010, 2011; adults: McNealy et al., 2006) and even species (e.g., monkey: Frost et al., 2015; Meyer & Olson, 2011). Seminal research found that even by 8 months old, infants were able to extract temporal regularities from both linguistic syllable sequences and nonlinguistic tone sequences (Saffran et al., 1996, 1999), with some studies providing evidence that even newborns are capable of auditory SL (e.g., Teinonen et al., 2009). However, this domain-general assumption has been challenged by more recent evidence of a dissociation in SL abilities across modalities and domains.

In terms of modality-specificity, SL of auditory regularities has been shown to be better than that of visual regularities (Conway & Christiansen, 2009; Emberson et al., 2011), and each modality exhibits distinct developmental trajectories among infants and children (Emberson et al., 2019; Raviv & Arnon, 2018). Specifically, it was found that auditory SL improved among 8- to 10-month-old infants, while visual SL at this age did not show a similar level of improvement (Emberson et al., 2019), indicating that the developmental patterns of SL across modalities is subserved by different processing mechanisms that have different developmental trajectories. Among older children aged 5 to 12 years, it was found that visual nonlinguistic SL significantly improved, while auditory linguistic SL remained stable (Raviv & Arnon, 2018); however, Shufaniya and Arnon (2018) reported that, in the same age group, both auditory and visual SL developed in the nonlinguistic domain, while only visual SL, but not auditory SL, developed in the linguistic domain (Shufaniya & Arnon, 2018). Additional evidence comes from research demonstrating that learners’ representations rely on modality-specific sensorimotor systems and cannot be separated from the perceptual features of the input (Conway & Christiansen, 2006). Regarding domain-specificity, Siegelman and Frost (2015) demonstrated that SL of linguistic and nonlinguistic input among adult learners involved distinct learning mechanisms, as there was no correlation between linguistic and nonlinguistic SL performance among adults, even within the same auditory modality. Differences in both accuracy and reaction times (RTs) also indicated the modality/domain specificity throughout development (Evans et al., 2009; Hu et al., 2024; Krogh et al., 2013; Qi et al., 2019). Taken together, these findings indicate that SL is not a unitary mechanism across domains but rather is constrained by domain-specific properties.

Current neuroimaging studies with adult populations have further confirmed that SL is indeed supported by both domain-general and domain-specific neural mechanisms (for review, see Batterink et al., 2019). The engagement of the inferior frontal gyrus (IFG) and the medial temporal lobe (MTL) has been consistently reported in SL across domains and modalities (for a recent review, see Forest et al., 2023), advocating for their involvement in domain-general SL processes. The IFG is activated during SL of both linguistic syllables (e.g., McNealy et al., 2006) and nonlinguistic tones (e.g., Abla & Okanoya, 2008), as well as during learning from both auditory and visual inputs (Karuza et al., 2013; McNealy et al., 2006; Turk-Browne et al., 2009). In the auditory modality, the tempofrontal network has been considered a domain-general mechanism during both linguistic and nonlinguistic SL (Assaneo et al., 2019; Farthouat et al., 2017; Moser et al., 2021; Orpella et al., 2022). Lesion studies have also revealed the role of the MTL in SL of both linguistic (syllable) and nonlinguistic (tone) regularities, as well as across auditory (syllable and tone) and visual modalities (shapes and scenes; Schapiro et al., 2014). However, other studies have failed to replicate this finding, indicating the role of the MTL in SL is less clear than previously reported (Covington et al., 2018; Wang et al., 2023).

In addition to these domain- and modality-general brain regions, neuroimaging research has demonstrated the involvement of domain-specific regions during SL. Specifically, the higher-level auditory network of the superior temporal gyrus (STG) has been involved in auditory SL (Cunillera et al., 2009; Karuza et al., 2013; McNealy et al., 2006; Moser et al., 2021), while the higher-level visual network, such as the lateral occipital-temporal cortex, shows stronger activation during visual SL (Turk-Browne et al., 2009). Focused on the temporal cortex, Tremblay et al. (2013) directly compared the neural basis underlying auditory SL of linguistic syllable sequences and nonlinguistic naturalistic bird sound sequences among adults, finding that activity in the bilateral medial transverse temporal sulcus and the right medial transverse temporal gyrus differed between highly structured and less structured tone sequences but not between highly and less structured syllable sequences, although both regions showed significant activation to both types of stimuli (Tremblay et al., 2013). Similarly, Schneider et al. (2024) found that, within the widely acknowledged language network (Fedorenko et al., 2010), the left posterior temporal gyrus was significantly activated during linguistic SL, but not during nonlinguistic SL. These results suggest that activation within the left posterior temporal gyrus during SL is specific to the processing of linguistic, but not nonlinguistic, regularities. These findings thus advocate for a dual model in which SL relies on domain-general learning mechanisms but is constrained by domain-specific processes (Batterink et al., 2019; Conway, 2020; Frost et al., 2015). This dual model gives credence to a distributed network of learning, wherein there are interactions between domain-general and domain-specific mechanisms.

Despite a wealth of research on the neural basis of SL in adult populations, relatively little is known about the neural basis of SL among developing children (Finn et al., 2019; McNealy et al., 2010, 2011). Adopting the same paradigm as implemented with adults, McNealy and colleagues revealed neural activation in bilateral superior temporal cortices (STC) and the left IFG among 6-, 10- and 13-year-old children when learning regularities embedded in structured syllable sequences (McNealy et al., 2010, 2011). Importantly, while neural activation in adults was left-lateralized (McNealy et al., 2006), 6-year-old children showed a right-lateralized distribution and 10- and 13-year-old children showed a bilateral distribution (McNealy et al., 2010, 2011). Moreover, the 6-year-old group showed more diffuse patterns of activation across the brain, including activation in the right transverse temporal gyrus, right insula, right precentral gyrus and left paracentral lobule. The ongoing shift in lateralization and more diffuse activation pattern throughout childhood indicate children do not necessarily activate the same neural regions as adults during SL, which necessitates further exploration.

In sum, behavioral evidence demonstrates that SL is available extremely early among infants and children but may be differentially constrained by domain-specific mechanisms (Hu et al., 2024). Limited neural evidence suggests that these behavioral differences in auditory SL in childhood may be subserved by ongoing neural development (McNealy et al., 2010, 2011; for review, Forest et al., 2023); however, no study to our knowledge has directly compared the neural basis of auditory SL across domains in the developing brain. To address this gap in the literature, the present study aims to directly compare the neural basis involved in auditory linguistic and nonlinguistic SL among children of 5–7 years old, an age range which has been shown to demonstrate differences in activation during auditory SL compared to older children and adults (McNealy et al., 2011), using a data-driven fMRI approach that accounts for individual differences in brain activity. Similar to McNealy et al. (2010, 2011), the present study focuses on the auditory modality considering the importance of auditory SL in literacy and language development (Qi et al., 2018). Children in the current study were exposed to interwoven sequences of structured and random syllables (i.e., linguistic) and tones (i.e., nonlinguistic; Schneider et al., 2020, 2024) as their neural data was collected using fMRI. The purpose of the present study is twofold: (1) identify the functional neural profiles of auditory SL in the developing brain for both linguistic (i.e., syllable) and nonlinguistic (i.e., tone) inputs; (2) identify whether linguistic and nonlinguistic auditory SL share a common neural architecture. Such an investigation can provide us with a better understanding of how SL is constrained by domain/modality-specific input among developing children.

## MATERIALS AND METHODS

### Participants

Thirty children ages 5–7 years (*M*_age_ = 6.45, *SD*_age_ = 1.05, females = 19) from the mid-Atlantic region of the United States participated in the current study. All participants were right-handed, monolingual English speakers, with no history of neurological disorder or developmental delay based on parental report. The study was approved by the Institutional Review Board at the University of Delaware and was in compliance with the Declaration of Helsinki.

### Auditory Statistical Learning Task

All stimuli and tasks utilized in the current study were modified from Schneider et al. (2020).

#### Stimuli

Syllable stimuli were constructed from 12 English consonant-vowel syllables (pi, pa, pu, ti, ta, tu, di, da, du, bi, ba, bu). All syllable stimuli were made using an artificial speech synthesizer and were recorded as separate files in a monotone female voice in Praat (Boersma & Weenink, 2017). Tone stimuli included 12 musical pure tones within the same octave (F, G, D, G#, C#, B, C, F#, D#, E, A, A#; a full chromatic scale starting from middle C). The duration of each syllable and tone was 460 ms with a 20 ms inter-stimulus interval based on previous studies utilizing this same presentation speed (i.e., Hu et al., 2024; Qi et al., 2019; Schneider et al., 2020).

During the exposure phase participants were exposed to sequences containing statistical regularities (i.e., structured blocks), no statistical regularities (i.e., random blocks), and silence (i.e., resting blocks). Structured blocks involved the presentation of four target triplets, which were created by concatenating the syllables into tri-syllabic pseudowords (pi-tu-bi, bu-pa-da, di-ba-pu, and ta-ti-du) and monotones into tri-tonal “melodies” (F#DE, ABC, C#A#F, and GD#G#). In other words, the three syllables of each syllabic pseudoword always appeared together in the structured syllable blocks and the three tones of each tri-tonal melody always appeared together in the structured tone blocks. The target syllable or tone to be tracked by participants was always the final sound within the triplet. All participants heard the same four target triplets (i.e., pseudowords and melodies) across structured blocks; however, the order of these were pseudo-randomized across participants to ensure there were no effects of order. This pseudo-randomization relied upon an algorithm which randomly chose a target triplet to be tracked, but included a rule wherein no identical target triplets were presented consecutively. Each of these target triplets lasted 1,440 ms. By contrast, random blocks contained the same 12 stimuli as presented in the structured blocks but were ordered in a pseudo-random way wherein no combinations of any three stimuli were repeated more than once. In other words, the algorithm randomly chose any single stimulus (i.e., syllable or tone) to be tracked; meanwhile, a rule was applied where any three continuous stimuli (i.e., three syllables or tones) only consecutively appeared once and therefore every combination of three continuous stimuli was a new and unique word. Resting blocks were silent.

**Figure 1.**
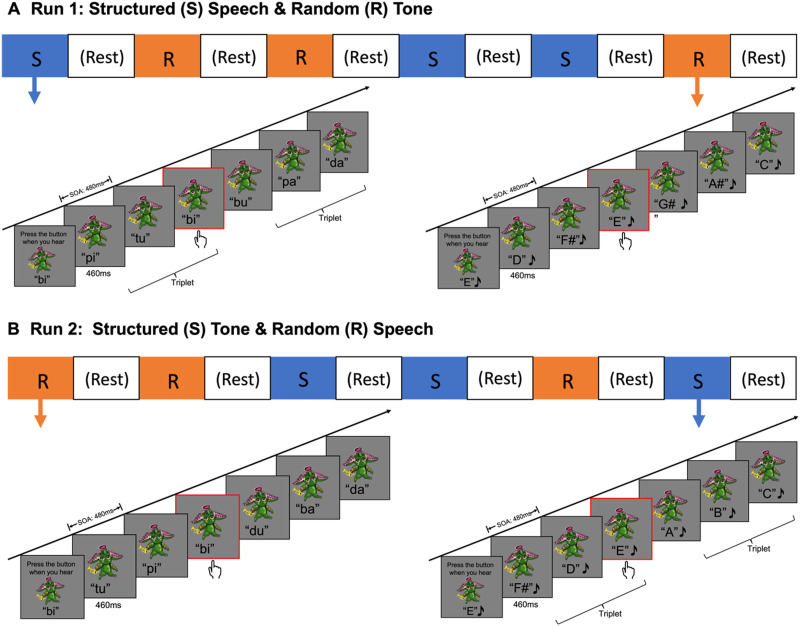
Overview of the auditory statistical learning fMRI task. Participants were exposed to (A) Run 1: Structured blocks each containing a 96-syllable sequence with the embedded 32 syllable triplets (4 syllable triplets with each presented 8 times), intermixed with three random blocks each containing a 96-tone sequence; and (B) Run 2: Random blocks each containing a 96-syllable randomly ordered sequence, intermixed with three structured blocks each containing a 96-tone sequences with 32 tone triplets (4 tone triplets with each presented 8 times). Participants were instructed to alternate between tracking a specific syllable (e.g., /bi/) and tracking a specific tone (e.g., “E”) in these sequences by pressing a button. Runs were counterbalanced across participants. *Note*: only the alien image, but not the spelling of the syllables, was present on the screen during the task. *Abbreviation*: SOA = stimulus onset asynchrony.

Three structured blocks and three random blocks were concatenated in a random order, with a resting block inserted after each block (totaling 6 resting blocks), to create one run of auditory stimuli (Figure 1). To maximize learning of the structured sequences, the random blocks within each run contained a different domain from the structured blocks (i.e., Figure 1A: syllable structured blocks were presented together with tone random blocks in one run; Figure 1B: syllable random blocks were presented together with tone structured blocks in the other run). Participants each completed two runs of the auditory SL task, the order of which was counterbalanced across participants. Each run lasted about 4 min and 36 s. In the run containing structured syllable blocks intermixed with random tone blocks, each structured syllable block contained four target syllable pseudowords, each of which was repeated 8 times within each block for a total of 24 presentations in the run (3 blocks). Each random tone block contained 96 pseudo-randomly ordered tones (total of 288 tones in the run). In the run with structured tone blocks intermixed with random syllable blocks, each structured tone block contained four target tone melodies, each of which was repeated 8 times within each block for a total of 24 presentations in the run (3 blocks). Each random syllable block contained 96 pseudo-randomly ordered syllables (total of 288 syllables in the run).

#### Procedure

During the exposure phase, tones and syllables were presented one at a time while children viewed a screen with a fixed image of an alien in the center of the screen. All auditory stimuli were presented to children using an MRI Noise Guard Headset (Z & Z Medical). Children also wore earplugs as an additional measure to protect their hearing. To ensure children could hear the stimuli, they completed a training phase. This training phase was initiated by first introducing the child to an alien before the exposure phase and told that they were going to listen to our alien friend, Klaptoo, speak a language and sing a song. They were then instructed to press the button in their left/right hand (counterbalanced across participants) on a button response pad (Cambridge Research Systems) whenever they heard the sound they were instructed to listen for. Children were randomly assigned to track one of four syllables (bi, da, pu, or du) and one of four tones (E, C, F, G#) in the third position of a triplet throughout both the training and exposure phase. For example, the participant may be asked to track /da/, which appeared only in the triplet “bu-pa-da.” During this training phase, the researcher tracked children’s responses to stimuli. After the training phase, if children did not accurately identify the target 60% of the time, or reported being unable to hear the stimuli, the audio volume was adjusted, and the training phase was re-initiated. Thus, the sound level was adjusted for each child. After the training phase, children began the exposure phase, in which RTs were recorded from these responses.

### Neuroimaging Data

The boilerplate text included in the Anatomical Data Preprocessing and Functional Data Preprocessing sections was automatically generated by fMRIPrep with the express intention that users should copy and paste this text into their manuscripts unchanged. It is released under a CC0 license.

#### MRI acquisition

Data were acquired on a Siemens 3T Magnetom Prisma scanner with a 64-channel phased array head coil at the Center for Brain and Biomedical Imaging at the University of Delaware. Prior to functional imaging, whole-head, high-resolution structural images, including a T1-weighted, magnetizations-prepared rapid gradient-echo (MPRAGE) anatomical volume (repetition time [TR] = 2,500 ms, echo time [TE] = 2.9 ms, inversion time = 1,070 ms, flip angle = 8.0°, voxel resolution = 1.0 mm isotropic, field of view [FOV] = 256 × 256, 176 sagittal slices) and a T2-weighted anatomical volume (TR = 3,200 ms, TE = 565 ms, flip angle = 2.0°, voxel resolution = 1.0 mm isotropic, FOV = 256 × 256, 32 sagittal slices) were collected.

Functional data were acquired using simultaneous T2*-weighted echo-planar imaging with multiband scans acquisition (Feinberg et al., 2010; Moeller et al., 2010; Setsompop et al., 2012) with the following acquisition parameters: TR = 800 ms, TE = 32 ms, flip angle = 61°, FOV = 210 × 210 mm, acceleration factor = 6. Across both runs, which included the syllable SL and tone SL tasks, required 60 adjacent slices in an interleaved order with 2.5 mm slice thickness resulting in an in-plane resolution of 2.5 × 2.5 × 2.5 mm^3^.

#### fMRI preprocessing

Functional and anatomical data were first converted using HeuDiConv (Halchenko et al., 2020) and then organized using the Brain Imaging Data Structure (BIDS; Gorgolewski et al., 2016). Preprocessing was completed using fMRIPrep (Version 1.3.1; Esteban et al., 2019; RRID:SCR_016216). fMRIprep combines methodology from AFNI (Cox & Hyde, 1997), ANTs (Version 2.2.0; Avants et al., 2008), FreeSurfer (Version 6.0.1; Dale et al., 1999), FSL (Version 5.0.9; Zhang et al., 2001), and Mindboggle (Klein et al., 2005, 2017) to provide scientifically rigorous and reproducible data for use in data analysis. fMRIPrep was first conducted on anatomical data only, described in more detail below. All functional data was then preprocessed based on these corrected T1 images.

#### Anatomical data preprocessing

The T1-weighted (T1w) image was corrected for intensity non-uniformity (INU) with N4BiasFieldCorrection (Tustison et al., 2010), distributed with ANTs (Version 2.2.0; Avants et al., 2008; RRID:SCR_004757), and used as a T1w-reference throughout the workflow. The T1w-reference was then skull-stripped with a Nipype (Gorgolewski et al., 2011) implementation of the antsBrainExtraction.sh workflow (from ANTs), using OASIS30ANTs as the target template. Brain tissue segmentation of cerebrospinal fluid (CSF), white matter (WM) and gray matter (GM) was performed on the brain-extracted T1w using FAST in FSL (Version 5.0.9; Zhang et al., 2001; RRID:SCR_002823). Brain surfaces were reconstructed using recon-all in FreeSurfer (Version 6.0.1; Dale et al., 1999; RRID:SCR_001847), and the brain mask estimated previously was refined with a custom variation of the method to reconcile ANTs-derived and FreeSurfer-derived segmentations of the cortical GM of Mindboggle (Klein et al., 2017; RRID:SCR_002438). Volume-based spatial normalization to one standard space (MNI152NLin2009cAsym) was performed through nonlinear registration with antsRegistration, using brain-extracted versions of both T1w reference and the T1w template. ICBM 152 Nonlinear Asymmetrical template (Version 2009c; Fonov et al., 2009; RRID:SCR_008796; TemplateFlow ID: MNI152NLin2009cAsym) was selected for spatial normalization.

#### Functional data preprocessing

For each of the two functional runs per subject, the following preprocessing was performed. First, a reference volume and its skull-stripped version were generated using a custom methodology of fMRIPrep. The blood oxygen-level dependent (BOLD) reference was then co-registered to the T1w reference using bbregister (FreeSurfer), which implements boundary-based registration (Greve & Fischl, 2009). Co-registration was configured with 6 *df* to account for distortions remaining in the BOLD reference. Head-motion parameters with respect to the BOLD reference (transformation matrices and six corresponding rotation and translation parameters) are estimated before any spatiotemporal filtering using mcflirt (FSL Version 5.0.9; Jenkinson et al., 2002). BOLD runs were slice-time corrected using 3dTshift from AFNI 20160207 (Cox & Hyde, 1997; RRID:SCR_005927). The BOLD time-series data were resampled to surfaces on the following space: fsaverage5. The BOLD time series (including slice-timing correction when applied) were resampled onto their original, native space by applying a single, composite transform to correct for head-motion and susceptibility distortions. These resampled BOLD time series will be referred to as *preprocessed BOLD in original space*, or just *preprocessed BOLD*.

The BOLD time series were resampled into standard space, generating a preprocessed BOLD run in MNI152NLin2009cAsym space. First, a reference volume and its skull-stripped version were generated using a custom methodology of fMRIPrep. Several confounding time series were calculated based on the preprocessed BOLD: framewise displacement (FD), DVARS, and three region-wise global signals. FD and DVARS are calculated for each functional run, both using their implementations in Nipype (following the definitions by Power et al., 2014). The three global signals are extracted within the CSF, the WM, and the whole-brain masks. Additionally, a set of physiological regressors were extracted to allow for component-based noise correction (CompCor; Behzadi et al., 2007). Principal components are estimated after high-pass filtering the preprocessed BOLD time series (using a discrete cosine filter with 128 s cutoff) for the two CompCor variants: temporal (tCompCor) and anatomical (aCompCor). tCompCor components are then calculated from the top 5% variable voxels within a mask covering the subcortical regions. This subcortical mask is obtained by heavily eroding the brain mask, which ensures it does not include cortical GM regions. For aCompCor, components are calculated within the intersection of the aforementioned mask and the union of CSF and WM masks calculated in T1w space, after their projection to the native space of each functional run (using the inverse BOLD-to-T1w transformation). Components are also calculated separately within the WM and CSF masks. For each CompCor decomposition, the *k* components with the largest singular values are retained, such that the retained components’ time series are sufficient to explain 50% of variance across the nuisance mask (CSF, WM, combined, or temporal). The remaining components are dropped from consideration. The head-motion estimates calculated in the correction step were also placed within the corresponding confounds file. The confound time series derived from head motion estimates and global signals were expanded with the inclusion of temporal derivatives and quadratic terms for each (Satterthwaite et al., 2013). Frames that exceeded a threshold of 0.5 mm FD or 1.5 standardized DVARS were annotated as motion outliers. All resamplings can be performed with a single interpolation step by composing all the pertinent transformations (i.e., head-motion transform matrices, susceptibility distortion correction when available, and co-registrations to anatomical and output spaces). Gridded (volumetric) resamplings were performed using antsApplyTransforms, configured with Lanczos interpolation to minimize the smoothing effects of other kernels (Lanczos, 1964). Non-gridded (surface) resamplings were performed using mri_vol2surf (FreeSurfer).

#### Whole-brain univariate analysis

First-level statistical analyses were carried out using FEAT (fMRI Expert Analysis Tool; Woolrich et al., 2001). For each individual run, parameter estimates for structured and random syllable/tone relative to baseline, as well as for contrasts of interest, were calculated. For the within-subject higher-level analysis, we combined data across runs by merging runs 1 and 2 together within FEAT for each participant. Specifically, we took parameter estimates from the first-level analysis of the structured speech from Run1 and compared it to the parameter estimates of the random speech in Run 2, and we took parameter estimates of the random tone from Run 1 and compared it with the structured tone in Run 2. Motion regressors were not included in the model. Given this analysis was exploratory in nature, we elected to compute group-level means for each contrast of interest using a less conservative fixed effect model (Beckmann et al., 2003). All *z*-statistic (Gaussianized time/frequency) images were thresholded at a cluster-forming threshold of *z* > 2.3 and a corrected cluster-level threshold of *p* = 0.05 (Worsley, 2012).

#### Group-constrained subject-specific analysis

Because understanding whether there are shared neural mechanisms underlying auditory SL depends critically on investigating functional activation within individual subjects, and considering the substantial individual differences that exist in SL (Siegelman et al., 2017), we also performed group-constrained subject-specific (GCSS) analyses designed to account for intrasubject variability (Fedorenko et al., 2010; Julian et al., 2012; Scott & Perrachione, 2019). With these analyses, we defined probabilistic regions of interest, or *parcels*, which were then used to constrain our individual subject analyses. We used these parcels to measure patterns of similarity across tasks within individuals.

We generated a set of parcels for each contrast of interest. In each case we thresholded the data at *p* < 0.01 and binarized each subjects’ contrast maps (structured > random, random > structured) in both the syllable and tone conditions. We then summed each set of maps to obtain a probability map. The probability map was smoothed with a Gaussian kernel of 6 mm full-width half maximum and further thresholded to include only voxels in which more than half (at least 60%) of participants showed significant activation. This smoothed map was then segmented into parcels algorithmically through two steps: (1) identifying all local maxima in the map; and (2) “growing” the parcels around these local maxima using a watershed algorithm (Meyer, 1991), which extends the parcel to adjacent voxels until the edge reaches a zero-valued voxel or a local minimum with SPM-SS toolbox (Fedorenko et al., 2010) We imposed a constraint that local maxima must be at least three voxels (or 1 cm) apart.

#### Univariate conjunction analysis

In order to identify regions within individuals that show significant activation for both linguistic and nonlinguistic auditory SL, we used the same approach to identify parcels based on individual conjunction maps. First, each structured > random contrast map for both tasks were voxel-wise thresholded at *p* < 0.01 and binarized. Then, for each subject, we computed the conjunction of their two binarized contrast maps. This resulted in one conjunction map per subject, which was then used to locate common conjunction areas across participants using a similar parcellation technique as described above, in the Whole-Brain Univariate Analysis section. Harvard–Oxford cortical and subcortical structural atlases were used to identify brain regions.

#### Local pattern similarity analysis

To ensure thorough investigation of neural patterns shared during linguistic and nonlinguistic SL, we conducted a local pattern similarity analysis (LPSA). This technique is designed to identify brain regions that support similar functions during different tasks, regardless of the exact level of activation (Scott & Perrachione, 2019). For this analysis, unsmoothed functional structured > random contrast maps from each task were compared. These maps underwent the same preprocessing and first-level analysis steps as the data discussed so far, except that they were not spatially smoothed or thresholded. To determine whether the pattern of activity in each SL parcel reflected similar task engagement during linguistic and nonlinguistic SL, we computed Pearson correlation coefficients between the syllable and tone contrast images from structured > random SL across all voxels in each parcel, within individual subjects. We assessed the significance of these correlations across our participants under a null hypothesis in which unrelated patterns of activity had a correlation of zero.

Next, correlations between the contrast maps of structured > random linguistic and nonlinguistic SL were computed across the whole brain using a 3-voxel radius searchlight for each subject. The searchlight is centered on each voxel in the brain and the Pearson correlation is computed between contrast maps for voxels falling within the sphere. The center voxel is then assigned the value of the resulting correlation coefficient, thus constructing a map of local correlations between tasks for each subject. The correlation maps were then Fisher-transformed and normalized resulting in a *z*-score correlation map for each subject. These maps were then combined across subjects using the GCSS to form parcels representing common regions with high pattern similarities across subjects. *Z*-scored correlation maps were thresholded at *z* = 2.3 (*p* < 0.01) and parcels with 60% or greater participants were chosen for this study. We report parcels that were significant at a threshold of 50% and 70% or more of participants in Supplementary Table 3 in the Supporting Information, available at https://doi.org/10.1162/nol_a_00156. The Harvard–Oxford cortical and subcortical structural atlases were used to identify brain regions.

## RESULTS

### Auditory SL Behavioral Performance

All behavioral analyses for the current study were conducted in R (RStudio Version 2023 12.1+402; R Core Team, 2012; RStudio Team, 2020). Similar to Schneider et al. (2020) and Hu et al. (2024), mean RT was computed as the average time it took for a participant to press the button for each target syllable or tone. In this analysis the button press had to occur in the time window of the stimulus onset (0 ms) and one stimulus after (+960 ms) the target to be considered a valid response. Eleven participants in the syllable condition and 15 participants in the tone condition were removed from the RT analysis, as they did not have enough valid key presses (<6 trials) during the exposure phase. This threshold of <6 trials was based on similar studies of SL in children (Hu et al., 2024; Schneider et al., 2020). Considering the implicit nature of SL, participants should be able to extract structural regularities from the input despite poor behavioral performance and were therefore included in all neuroimaging analyses. Behavioral RT analyses were conducted with 19 participants (*M*_age_ = 6.51 yr, *SD*_age_ = 1.06 yr, *n* females = 13) in the syllable condition and 15 participants in the tone condition (*M*_age_ = 6.35 yr, *SD*_age_ = 1.07 yr, *n* females = 11). The number of trials retained per participant, and whether they were included in the behavioral analysis or removed, is reported in Supplementary Table 1.

Similar to Siegelman et al. (2018), we log-transformed the RT for each subject to account for variance in baseline RTs of different subjects. Results showed that there was no significant difference in the mean RT to the target syllable across the entire syllable exposure phase between structured (*M* = 422.22 ms, *SD* = 156.68 ms; *M*_log_ = 5.99 ms, *SD*_log_ = 0.35 ms) and random conditions (*M* = 459.09 ms, *SD* = 172.09 ms; *M*_log_ = 6.06 , *SD*_log_ = 0.37; *t*(17) = −1.01, *p* = 0.33). The same pattern was observed in the tone condition, with no significant differences in mean RT to tone targets across the entire tone exposure phase between structured (*M* = 397.29 ms, *SD* = 167.73 ms; *M*_log_ = 5.90, *SD*_log_ = 0.44) and random (*M* = 375.37 ms, *SD* = 107.56 ms; *M*_log_ = 5.89, *SD*_log_ = 0.28; *t*(13) = 0.37, *p* = 0.72) conditions. In addition, the mean RT difference (structured − random) does not differ between syllable and tone tasks (*t*(15) = −0.48, *p* = 0.64). To evaluate whether learning varied over the course of the exposure period, we extracted RT responses within each block and conducted paired *t* tests to compare each structured and random block within syllable and tone conditions. For both syllable (Supplementary Table 2) and tone (Supplementary Table 3) conditions, no differences in the log-transformed RT were found between structured block 1 and random block 1 (syllable: *t*(12) = 0.73, *p* = 0.48; tone: *t*(6) = −1.24, *p* = 0.26), between structured block 2 and random block 2 (syllable: *t*(12) = 0.14, *p* = 0.89; tone: *t*(9) = −1.25, *p* = 0.24), and between structured block 3 and random block 3 (syllable: *t*(16) = −0.24, *p* = 0.81; tone: *t*(8) = 0.79, *p* = 0.45).

To establish whether all participants included in the analysis performed the task at above-chance levels, we also computed *A*′ values as this is a measure of the sensitivity for correctly detecting a stimulus based on hit and false alarm rates (Aaronson & Watts, 1987; Grier, 1971; Pallier, 2002). The *A*′ was calculated with the aprime function in the psycho package in R (Makowski, 2018; formulas used by the aprime function to calculate *A*′ are: when false alarm (fa) rate < hit rate (hit): *A*′ = 1/2 + ((hit − fa) * (1 + hit − fa) / (4 * hit * (1 − fa))); when false alarm rate > hit rate: *A*′ = 1/2 − ((fa − hit) * (1 + fa − hit) / (4 * fa * (1 − hit))).). An *A*′ near 1.0 indicates good discriminability, while a value near 0.5 indicates chance performance. Similar to our mean RT analysis, 11 participants in the syllable condition and 15 participants in the tone condition were removed from the *A*′ analysis, as they did not have enough valid key presses (<6 trials) to calculate hits during the exposure phase. For responses made in the time window of 0–960 ms after stimulus presentation, the mean *A*′ value on the auditory, linguistic statistical learning task (syllable) was 0.70 (*SD* = 0.10) and the mean *A*′ value on the auditory, nonlinguistic statistical learning task (tone) was 0.67 (*SD* = 0.06). No participants had an *A*′ value of less than 0.50 on either the syllable task (range: 0.55–0.89) or the tone task (range: 0.51–0.76). Individual *A*′ values are provided in Supplementary Table 4. Paired *t* tests showed that there were no significant detection differences between structured and random sequences in either the syllable or tone task. Specifically, no statistical difference was found between syllable structured (*M*(*SD*)_structured A′_ = 0.72(0.13)) and random sequences (*M*(*SD*)_random A′_ = 0.67(0.14); *t*(18) = 1.48, *p* = 0.16), or between tone structured (*M*(*SD*)_structured A′_ = 0.60(0.15)) and random sequences (*M*(*SD*)_random A′_ = 0.68(0.08); *t*(14) = −1.63, *p* = 0.13).

We also compared the *A*′s for each block within the syllable and tone conditions using paired *t* tests. For the syllable condition (Supplementary Table 5), the *A*′ in the structured syllable block 1 was marginally higher compared to the random syllable block 1 (*t*(17) = 1.80, *p* = 0.09), while no differences in *A*′ were found between structured syllable block 2 and random syllable block 2 (*t*(15) = −0.16, *p* = 0.87) and between structured syllable block 3 and random syllable block 3 (*t*(17) = −0.04, *p* = 0.97). For the tone condition (Supplementary Table 6), *A*′ was marginally higher in the structured tone block 2 compared with the random tone block 2 (*t*(13) = −2.21, *p* = 0.05), while no differences were found between structured and random tone block 1 (*t*(14) = −1.27, *p* = 0.22) and block 3 (*t*(12) = 1.30, *p* = 0.22).

### Group-Level Univariate Analysis

To evaluate the quality of our fMRI data we obtained the FD values of each participant as listed in Supplementary Table 7. The FD value reflects the motion contamination of the data, with higher FD values indicating more motion artifacts. It is suggested that the FD threshold should be established and reported based on subject-specific data (Power et al., 2015). In our present study, we extracted the FD value of each subject and each run. We then calculated the mean FD values of both runs for each participant. Three participants with FD values 1.5 *SD* above the group FD mean (0.64) were excluded from all neuroimaging analyses. Therefore, all fMRI analyses were conducted on the remaining 27 participants (*M*_age_ = 6.45 yr, *SD*_age_ = 1.03 yr, *n* females = 18).

**Figure 2.**
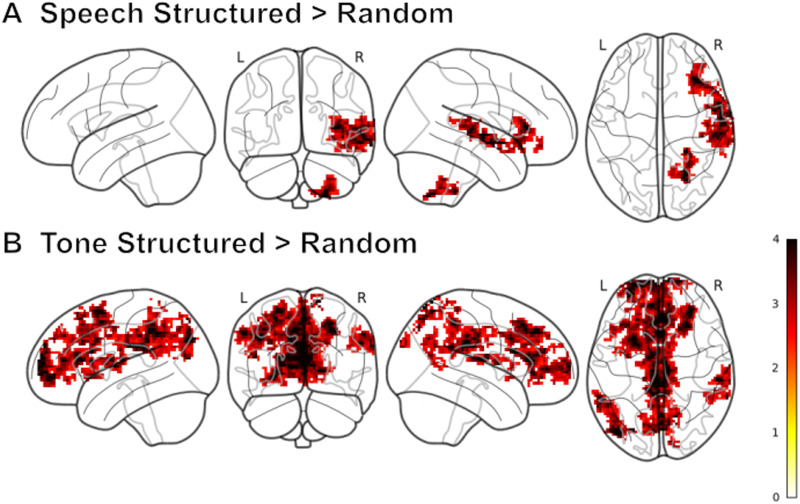
Whole-brain univariate analysis. Group mean *z*-statistic maps of structure > random contrast of both (A) syllable and (B) tone tasks with a cluster-based threshold of *z* > 2.3 and *p* < 0.05. The color bar represents the *z*-normed (*z*-stat) mean activity (*β*) across participants at each voxel.

With a cluster-based threshold of *z* > 2.3, traditional group-level univariate analysis showed that processing of syllable structured versus random sequences activated the right superior temporal gyrus (STG) and right cerebellum (Figure 2A). Processing of tone structured versus random sequences engaged the left middle frontal gyrus (MFG), left superior frontal gyrus (SFG), right supramarginal gyrus, posterior/angular gyrus (AG), and cingulate gyrus (CG; Figure 2B). Traditional conjunction analyses failed to reveal significant activation across tasks (syllable structured > random contrast and tone structured > random contrast).

### Group-Constrained Subject-Specific Analysis

**Figure 3.**
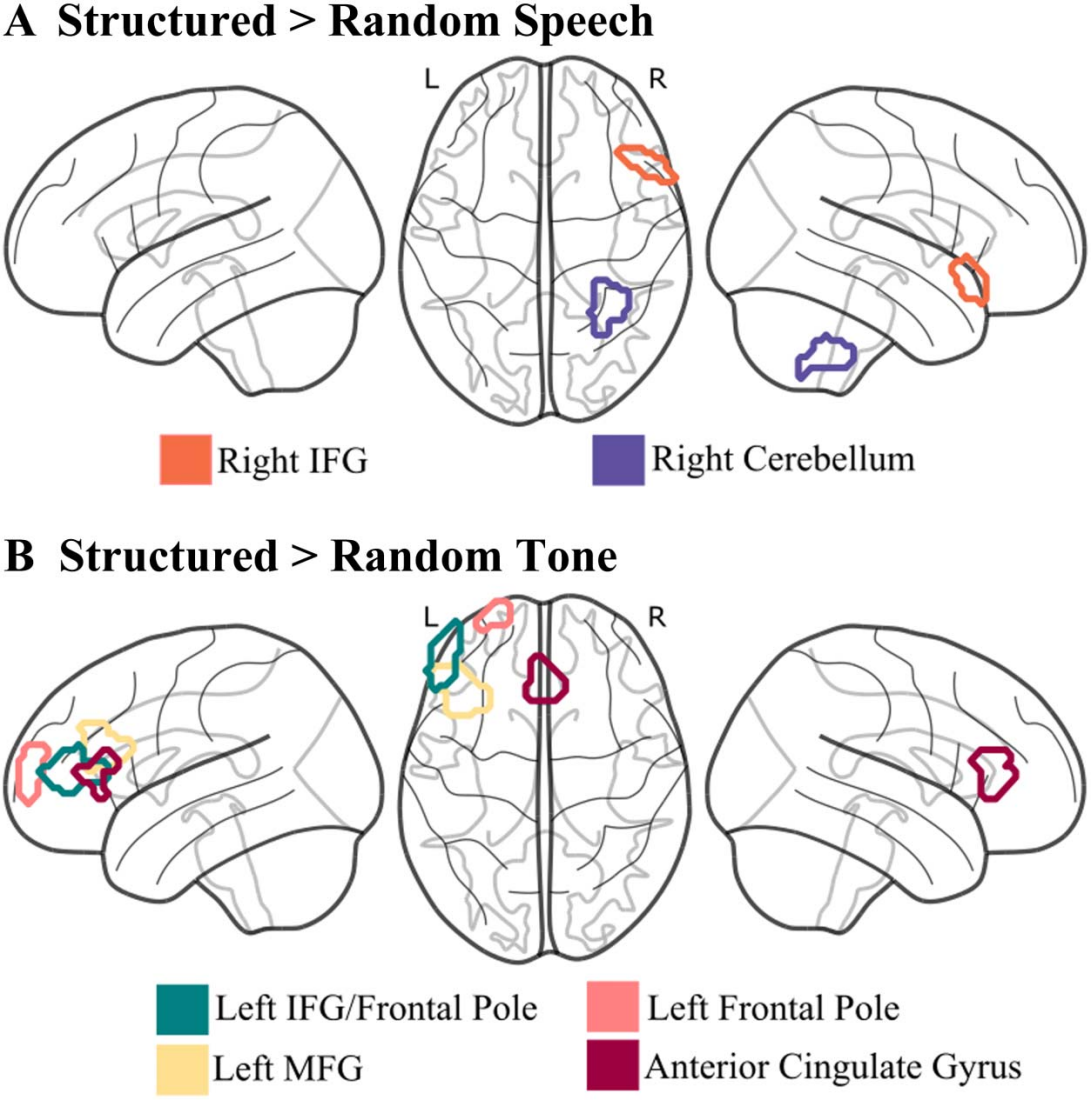
Group-constrained subject-specific analysis for processing of structured versus random regularities across domains. Parcels represent significant activation (*z* > 2.326) for structured > random (A) syllable and (B) tone regularities among more than 60% of participants. IFG = inferior frontal gyrus; MFG = middle frontal gyrus.

To identify the functional neural profiles of auditory SL in the developing brain during processing of linguistic and nonlinguistic regularities, we generated a set of parcels for each contrast of interest. Statistical maps were thresholded at *p* < 0.01, and only parcels that were active among 60% or more participants are reported. We also report the results of this parcellation at a threshold of 50% and 70% or more of participants (Supplementary Table 8). This GCSS parcellation analysis revealed two parcels that were sensitive to processing of structured versus random syllable sequences (structured > random syllable): right IFG (*N* = 18, 60%; Cohen’s *d* = 0.28; power = 0.29) and the right cerebellum (*N* = 18, 60%; Cohen’s *d* = 0.11; power = 0.08; Figure 3A). In the tone condition, four parcels emerged as significant when processing structured versus random tones (structured > random tone): left IFG (*N* = 17, 63%; Cohen’s *d* = 0.51; power = 0.72), left FP (*N* = 17, 63%; Cohen’s *d* = 0.38; power = 0.47), left MFG (*N* = 18; 67%; Cohen’s *d* = 0.38; power = 0.47), and anterior cingulate gyrus (ACG; *N* = 20, 74%; Cohen’s *d* = 0.73; power = 0.96; Figure 3B).

Although not the main goal of the current study, we also examined random > structured contrasts for both syllable and tone tasks. Five parcels were sensitive to processing of random versus structured syllable sequences (random > structured syllable): left IFG (*N* = 17, 63%), left MFG (*N* = 19, 70%), right MTG (*N* = 20, 74%), left superior parietal lobule (SPL; *N* = 21, 78%), and left AG (*N* = 17, 63%; Supplementary Figure 1A). Two parcels at left STG (*N* = 18, 67%) and left SFG (*N* = 17, 63%) displayed sensitivity to processing of random versus structured tone sequences (Supplementary Figure 1B).

### Lack of Spatial Conjunction Across Domains During Auditory SL in Children

In order to identify regions within individuals that showed significant activation for both linguistic and nonlinguistic auditory SL, we computed individual conjunction maps using the same GCSS analysis described above (see Materials and Methods). This analysis allows us to locate common regions of conjunction across tasks and participants. We hypothesized that brain regions engaged by both tasks during processing of structured > random sequences may underlie a domain-general mechanism of auditory SL. Our results showed that no parcels were significantly relevant for both syllable and tone processing across 60% or more of participants. These same results held when adjusting this threshold to 50% and 70% or more of participants (Supplementary Table 4).

Given the lack of neural conjunction between tasks, and lack of behavioral results, we sought to further evaluate the consistency of our neuroimaging data by examining the strength of the correlation between neural activation both within and between tasks. The purpose of this analysis was to compare whether within task data showed significantly higher correlations or consistency across individuals than the between-task data. To accomplish this, we extracted the top 10% of voxels from the subject’s statistical T-map within each parcel (Scott & Perrachione, 2019) from each structured block: three structured blocks from the syllable condition (run 1) and three structured blocks from the tone condition (run 2). We then calculated the correlation of activation patterns both within tasks between structured blocks of the same stimulus type and between tasks for each structured block in each parcel. For the within task comparison, we calculated the correlation of activation pattern between structured blocks 1, 2, and 3 of syllable structured sequences within our two syllable parcels, and we calculated the correlation of activation pattern between structured blocks 1, 2, and 3 of tone structured sequences within our four tone parcels. Supplementary Tables 9 and 10 provide the within-task correlation between structured blocks, with all parcels demonstrating significant within-task correlations except for the right cerebellum in the syllable task and left MFG in the tone task. For the between task comparison, we calculated the correlation of activation pattern between tasks for each structured block in all six parcels. As can be seen in Supplementary Table 11, only the third blocks between tasks were correlated in the left MFG and the second blocks between tasks were correlated in the left IFG. Although we could not directly compare within- and between-task correlations, we found substantially more correlations across blocks within tasks, as compared to between tasks, speaking to the consistency of neural activation across blocks and individuals.

### Patterns of Neural Activation Within SL Parcels Are Not Similar Across Tasks in Individual Subjects

**Figure 4.**
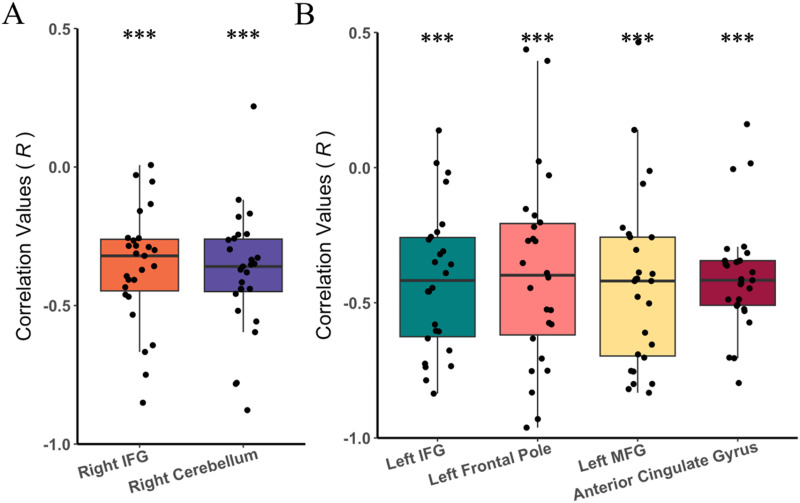
Correlation *r* values for activation pattern between syllable and tone contrasts within parcels identified as significant for processing of (A) syllable structured > random and (B) tone structured > random. Each point stands for each subject. ***p < 0.001.

The lack of spatial conjunction identified by univariate analyses does not necessarily indicate absence of co-activation across both tasks. Univariate analyses ignore the subtle activation pattern in local areas of the brain. Therefore, we used the LPSA approach described in Materials and Methods to determine whether activation patterns during processing of structured > random syllable and tone regularities were similar within parcels identified as significant in the GCSS analysis. We assessed the significance of these correlations across our participants under a null hypothesis in which unrelated patterns of activity had a correlation of zero. After correlating across all voxels within each parcel between syllable and tone tasks for each participant, one-sample *t* tests indicated significant negative correlations across tasks within all parcels (all *p* values = *p* < 0.001; Figure 4).

### No Common Activation Region Was Shared by SL Across Domains

We last sought to reveal more subtle correlations between patterns of activation across tasks in the whole brain, rather than identifying the most highly activated regions. To accomplish this, we implemented a whole-brain LPSA to assess the voxel-wise correlations across both tasks within each individual participant. Using a 3-voxel radius spherical searchlight, the local correlation coefficients between the two tasks were calculated for each centered voxel. We chose a more liberal threshold that is still appropriate for voxel-wise tests (*p* < 0.01) and focused on parcels with agreement among 60% or more of participants. Similar to our previous conjunction analyses, our results did not yield any parcel that was significantly relevant for both syllable and tone processing across 60% or more of participants. When plotting individual whole-brain correlation maps (Supplementary Figure 2), substantial heterogeneity across children was observed.

## DISCUSSION

The current study directly investigated the neural basis of auditory SL across linguistic and nonlinguistic domains to establish an understanding of how SL is constrained by domain/modality-specific features among developing children. With an RT window of 0–960 ms, no significant behavioral differences were observed between structured and random sequences in both the linguistic and nonlinguistic conditions. Our GCSS analysis revealed significant activity in the right IFG and right cerebellum during SL of structured versus random syllable sequences, and significant activity in the left IFG, left FP, left MFG, and ACG during SL of structured versus random tone sequences. Surprisingly, no spatial conjunction between brain regions was uncovered during SL of linguistic and nonlinguistic regularities. Although activity in the IFG was detected during SL of both syllables and tones, the lateralization of this activation differed across tasks. This distributed activation and lack of neural conjunction may be the result of the immature and heterogenous neurobiological organization of the developing brain or a domain-specific neural basis of SL among developing children (Brown, 2017).

In the current study, we failed to uncover behavioral differences between structured and random blocks, with many children being unable to meet the minimum of six valid responses. Children who completed the tasks, were able to detect the target syllables and tones reliably, as determined by *A*′ values; however, they were no better at detecting structured versus random sequences. Considering SL occurs rapidly, and is detectable as early as the first months of life (Saffran et al., 1996), the lack of behavioral differences is surprising. However, SL is commonly regarded as an implicit learning mechanism that can occur in the absence of consciousness or awareness (Batterink et al., 2015; Conway, 2020; Perruchet, 2019; Perruchet & Pacton, 2006), and neural differences are often observed in response to learning of statistical regularities even when behavioral differences are not present (children: McNealy et al., 2010, 2011; Scott-Van Zeeland et al., 2010; adults: McNealy et al., 2006). Karuza et al. (2014) proposed that a possible way to examine implicit SL when behavioral performance is not yet evident, is to compare neural activation between one condition where learning can happen (i.e., structured) and a condition where learning is not possible (i.e., random). The purpose of including this contrast is to isolate neural systems that are sensitive to tracking of statistical regularities. When comparing structured versus random sequences in the current study, we identified a number of brain regions that have been shown to be sensitive to implicit, auditory SL in past studies (McNealy et al., 2006, 2010, 2011). Specifically, among children 6–10 years old, McNealy et al. (2010, 2011) uncovered a number of similar brain regions, including IFG and STG, that were sensitive to these statistical patterns despite no differences in either accuracy or RTs during learning being reported (McNealy et al., 2010, 2011; see also Scott-Van Zeeland et al., 2010). This same pattern of results has also been reported in adults (McNealy et al., 2006).

It was surprising that there was no conjunction across linguistic and nonlinguistic domains in any region of the brain, despite probing this relationship using several different approaches (e.g., univariate analyses, GCSS, and LPSA). Our LPSA results demonstrate a significant negative correlation between linguistic and nonlinguistic tasks within each parcel generated by the GCSS analysis. This indicates that the more active a region was for processing of linguistic statistical regularities, the less active the region was during processing of nonlinguistic statistical regularities, and vice versa. This task-induced deactivation may occur in brain regions that are relevant to SL in the opposite domain in a manner that boosts neural processing efficiency and benefits task performance (Morita et al., 2019). This negative correlation was also observed in a recent study adopting the same SL task among adults (Schneider et al., 2024). Specifically, Schneider and colleagues found that the left posterior temporal gyrus showed significant activation during linguistic SL, but deactivation during nonlinguistic SL. Studies that have not examined SL have also revealed similar evidence of task-induced deactivation. For example, during a motor button-press task, activation in both the visual and auditory cortex were suppressed (Morita et al., 2019). Moreover, the deactivation in the visual cortex was associated with better motor performance (Morita et al., 2019). It was therefore proposed that cross-modal deactivation allows the brain to focus on precise motor performance and protect the brain from cross-modal distraction and interference from the irrelevant sensory modality (Morita et al., 2019). When situating the current findings in this past literature, we interpret deactivation in brain regions necessary for auditory SL across domains as representing a compensatory strategy, wherein the suppression of activation in a given region for the nontarget domain allows for the allocation of attentional resources for children to learn regularities in the target domain.

There are three alternative explanations for the lack of conjunction between linguistic and nonlinguistic domains. First, SL may be primarily constrained by domain-specific properties in children’s brains, with certain regions becoming more domain-general with age and experience. Among adults, the IFG in the frontal cortex is widely considered to support a modality/domain-general neural basis of SL, as it is engaged when processing inputs across a variety of modality/domain-specific perceptual/motor brain regions (Clerget et al., 2012; Conway, 2020). By contrast, the hierarchical organization of neural regions relevant for SL, such as the frontal cortex, are significantly less developed among children 7–9 years old (Supekar et al., 2009). Combining these findings together, it is possible that the frontal cortex of children is still immature and the domain-general processing functions of this region and others relevant for SL are still developing. It may be that SL becomes more domain general as the frontal cortex and subregions like the IFG develop as a function of natural brain maturation.

Second, it is possible that the lack of conjunction across domains is attributed to substantial heterogeneity due to extensive ongoing plasticity in children’s brains (Cui et al., 2020). The right, but not the left, lateralization of activation in the IFG and STG during SL of syllables in the current study, and substantial heterogeneity revealed by our whole-brain LPSA, supports this hypothesis. Past research has shown that both the left and right hemispheres are involved in language processing among 4- to 6-year-old children, with right hemisphere activation decreasing with age, resulting in a more left lateralized language network among adults (Olulade et al., 2020). These findings show not only that the right hemisphere is involved in language processing among developing children but also that the lack of conjunction across tasks may be attributed to right lateralization of brain during children’s brain development. However, our ability to make claims about the developmental trajectory of neural regions supporting SL is limited as we were unable to compare activation in 5- to 7-year-old children with older children and adults.

Third, the lack of conjunction between linguistic and nonlinguistic domains in the present study might be attributed to substantial variability present in children’s neuroimaging data. We predicted high, positive correlation coefficients among within-condition blocks, and low or insignificant correlation coefficients among between-condition blocks. However, our correlation analysis evaluating the consistency of data among blocks within each condition (Supplementary Table 5 for syllable condition and Supplementary Table 6 for tone condition), and between blocks across conditions (Supplementary Table 7) in each parcel did not fully support this prediction. Although more significant correlations were found in the within-condition correlations, the difference between within- and between-condition correlation patterns is difficult to interpret. This is because within-condition analysis examined the correlation among blocks 1, 2, and 3 within each condition, while between-condition analysis examined correlations for a single block (block 1, 2, or 3) across conditions. Considering that the parcels revealed in the present study are similar to those regions found in previous studies (McNealy et al., 2010, 2011), one possibility is that learning is a continuous process and unfolds over time, and therefore, the activation pattern in block 1 may differ from the activation pattern in block 3, resulting in potentially fewer within-condition correlations than anticipated. However, this overall lack of consistency may also reflect that the neuroimaging data collected in young children using the current tasks are somewhat noisy and unreliable, resulting in a lack of conjunction across tasks. Future studies of young children with more consistent neural data across blocks are still needed to examine the domain generality of the neural basis of SL among developing children.

Both linguistic and nonlinguistic tasks engaged the IFG. Consistently, the left IFG is thought to be critical in processing structured sequences, unifying information, computing statistical regularities, and forming structural representations, regardless of domain or modality (Petersson et al., 2012). Thus, prior studies propose that the left IFG underlies a domain-general neural basis of SL (Batterink et al., 2019). Specifically, activation of left IFG was found during linguistic SL (Cunillera et al., 2009; Karuza et al., 2013; McNealy et al., 2006), nonlinguistic SL (Abla & Okanoya, 2008), and visual SL (Turk-Browne et al., 2009) among adults. In children, greater activity in the left IFG was observed during processing of syllable triplets embedded in structured syllable sequence (McNealy et al., 2010).

In the present study, however, engagement of the IFG was contralateral across linguistic syllable and nonlinguistic tone SL, with linguistic SL activation in the right IFG, and nonlinguistic SL activation in the left IFG. The contralateral activation of this region across tasks could be largely attributed to the ongoing functional specialization of language specific brain regions in children (Olulade et al., 2020; Prat et al., 2023). Recent research has revealed that both the left and right hemisphere are equally involved in language processing early in life, although the contribution of the right hemisphere decreases throughout childhood (Olulade et al., 2020). Despite the left hemisphere long being considered the dominant hemisphere for more proficient language processing, activation of the right hemisphere is thought to be foundational for the acquisition of a new language (e.g., Prat et al., 2023; Qi et al., 2019; Qi & Legault, 2020). Accordingly, Goldberg and Costa (1981) and Prat et al. (2023) proposed that the right hemisphere is well-suited for detecting novelty and extracting features and rules from new linguistic information. In the present study, the SL of syllable sequences also requires the acquisition of new linguistic rules, similar to the acquisition of a new language, which may account for the right lateralization of the IFG. This differential lateralization also provides an explanation for the lack of convergence between SL of tone and syllable sequences.

Although we attribute the right lateralization of IFG activation during linguistic SL to right-lateralized language processing during early development, the functional conjunction between linguistic SL and language processing in children remains unknown. Schneider et al. (2024) recently conducted a similar SL task among adults to directly examine if SL shares a common neural basis with language processing, given the widely acknowledged role of SL as a fundamental construct for language development. Adopting similar GCSS and multivoxel pattern analyses among 12 brain regions involved in language processing (Fedorenko et al., 2010), the authors found that there was no activation of the IFG during linguistic SL among adults. It was proposed that different, but adjacent, subregions of the IFG might underlie linguistic SL and language processing. While the current study uncovered activation in the right IFG during linguistic SL, future research seeks to examine whether this same region is active during language processing in the developing brain, and whether lateralization differences during SL shift as a function of age.

Traditional univariate analyses implemented in the current study also highlight the role of the right STG during SL of structured versus random syllable sequences, which is in alignment with previous studies in children (McNealy et al., 2010, 2011). This finding is not particularly surprising considering the STG plays an important role in speech processing (Henin et al., 2021; Hickok & Poeppel, 2007; Leonard et al., 2015) and auditory working memory (Leff et al., 2009). There are, however, differences in the lateralization of STG activation across studies. Specifically, right and bilateral activations in the superior temporal cortices (STC) have been observed in children ages 6, 10, and 13 when learning regularities embedded in structured syllable sequences (McNealy et al., 2010, 2011). By contrast, left lateralized activation in the temporal cortex was primarily found among adults (Cunillera et al., 2009; Karuza et al., 2013; McNealy et al., 2006). Therefore, we speculate that activation of the right STG during auditory linguistic SL in the present study might be due to ongoing neural specialization in the developing brain, wherein the right hemisphere still plays an important role in linguistic information processing in childhood (Olulade et al., 2020).

The simultaneous activation of the right IFG and STG during SL of syllable regularities might be attributed to a functional link which exists between the frontal and temporal regions during SL, as revealed in previous studies in adults (for review, see Conway, 2020). Researchers have proposed that two systems in the brain, the sensory/perceptual network and frontal brain regions, work in a hierarchical fashion during SL (Henin et al., 2021; for review, see Conway, 2020). First, a variety of perceptual subregions are activated depending on the type of sensory input (e.g., temporal for auditory stimuli and inferotemporal cortex for visual stimuli; Fuster & Bressler, 2012). These perceptual systems process low-level sensory information with a short timescale, while the frontal system processes more complex, high-level information (Conway, 2020). In a recent study implementing an artificial grammar task, Henin and colleagues (2021) revealed early processing of lower-level units (i.e., syllables) in the STG and later processing of higher-order units (i.e., learned words) in the IFG. Consistent with this proposal, studies have revealed that activity in the IFG was associated with offline behavioral performance following exposure to statistical regularities (McNealy et al., 2010; Turk-Browne et al., 2009). The association between offline behavioral performance and activity in the IFG indicates that learned regularities during SL might be tracked and stored within the IFG. The current study demonstrated activity in both auditory regions of the STG and frontal regions of the IFG, which might indicate the hierarchical pathway of perceptual to high-level processing during SL is developing in children (Conway, 2020), although functional connectivity analyses are required to verify this prediction.

The bilateral activation in the IFG might also indicate the contribution of the audio-motor network during auditory SL (Assaneo et al., 2019; Farthouat et al., 2017; Moser et al., 2021; Orpella et al., 2022), given the IFG has been implicated in studies of audio-motor connectivity during processing of both linguistic and nonlinguistic regularities (Ohashi & Ostry, 2021; Palomar-Garcia et al., 2020). Assaneo et al. (2019) revealed a bimodal distribution of performance in a spontaneous synchronization of speech task (SSS test), in which participants were required to produce syllables while listening to a rhythmic syllable stream. Specifically, the high-synchrony participants aligned the syllable production with the rhythm of the perceived syllable stream, while the low-synchrony group remained impervious to the concurrent syllable rhythm. Furthermore, the high-synchrony group showed increased brain-to-stimulus synchronization over the frontal cortex and enhanced WM pathways connecting frontal and auditory regions, both of which supported better performance in the auditory SL task (Assaneo et al., 2019; Orpella et al., 2022). The tempofrontal network was also engaged during SL of tones (Farthouat et al., 2017; Moser et al., 2021), indicating that the audio-motor interface is a domain-general mechanism underlying SL. By contrast, a recent behavioral study showed that the auditory-motor mechanism during SL represents a domain-specific mechanism, with the speech-motor system (whispering) showing a stronger effect on speech SL than tone SL, while the hand-motor system (clapping) did not affect learning (Boeve et al., 2024). In our present study, both the linguistic syllable and nonlinguistic tone SL tasks resulted in activation of the right and left IFG, respectively. However, the domain-specificity of the IFG in the present study is not conclusive. On the one hand, it is possible that bilateral IFG activation is responsible for general learning of regularities; on the other hand, it is also possible that activation of the left IFG during processing of auditory, nonlinguistic regularities is specific to nonspeech motor processing, while activation of the right IFG during processing of auditory, linguistic regularities is specific to speech motor processing. Future research should directly examine the domain-specificity of IFG activation and the connectivity between the frontal and temporal cortex during linguistic and nonlinguistic SL.

The right cerebellum was also active during processing of structured versus random syllable sequences. Although the cerebellum is traditionally considered relevant for motor control, recent theories have implicated cerebellar function in nonmotor cognition of speech and language processing (e.g., Lesage et al., 2012; for review, see Mariën et al., 2014). This cross-domain role of the cerebellum might be due to the homogeneous cytoarchitecture of the cerebellum (Ramnani, 2006), as well as its GM properties—which have been linked to cognitive performance in a myriad of domains, like reading and working memory (Moore et al., 2017). Importantly, the cerebellum is proposed as a predictive machine (Stockert et al., 2021), making it relevant for extracting sequence order information and establishing internal models (Ito, 2006; Molinari et al., 1997). Specific to language, the cerebellum has been found to predict and encode the temporal structure of successive language events (Schwartze & Kotz, 2016; Schwartze et al., 2012). Taken from this understanding of the cerebellum’s role in linguistic processing and prediction of upcoming syllables in speech, our findings suggest the cerebellum may play a role in auditory linguistic SL in children. The role of cerebellum in SL, however, needs more exploration considering that the effect size and power observed in this region were relatively low.

The left FP, a region of the larger dorsolateral prefrontal cortex (DLPFC), showed stronger activation during processing of structured tone sequences compared to random tone sequences. Research in adults has demonstrated activation of the left FP during SL of tone sequences (Abla & Okanoya, 2008), and the current findings add evidence that this same region is relevant for SL of tone sequences in children. The necessity of the DLPFC during SL remains widely contested (Ambrus et al., 2020; Janacsek et al., 2015; Nydam et al., 2018; Prutean et al., 2021; Smalle et al., 2022). Disruption of the DLPFC has been found to promote structured sequence learning (Ambrus et al., 2020; Park et al., 2022; Smalle et al., 2022). The promotive effect that inhibiting the PFC has upon SL is considered a shift to model-free learning, as inhibition weakens access to model-based learning associated with previously acquired knowledge (Ambrus et al., 2020). By contrast though, there are studies which reveal activation of the DLPFC is integral for SL (Janacsek et al., 2015; Nydam et al., 2018). This paradox may be explained by research in adults, wherein predictions about upcoming regularities are rooted in prior experiences (Siegelman & Frost, 2015). These prior experiences and expectations may override new information, making it difficult for adults to learn new patterns, and therefore the inhibition of the DLPFC positively contributes to the acquisition of new structured patterns (Ambrus et al., 2020; Daikoku et al., 2021). By contrast, the DLPFC is not as mature among developing children as it is in adults (Friederici et al., 2017). Therefore, an appropriate level of prediction from the DLPFC during SL among children might be promotive for the formation and consolidation of new patterns through the interaction between the PFC and auditory cortex (Conway, 2020).

In addition, activity in the ACG was observed during SL of tone sequences. The ACG is a major neural hub supporting adaptation and learning from a rapidly changing environment (Rushworth et al., 2011). Prediction of likely events, signaling deviation between expected and observed events and monitoring task performance is one of the mechanisms through which the ACG supports learning from the changing environment (Alexander & Brown, 2019). Accordingly, in the present study, the ACG might support SL of regularities in tone sequences through continuous prediction and prediction error.

Last but not least, the activation in the hippocampus/MTG was not significant among 60% or more of participants. When adjusting this threshold, we did find significant activation of the left hippocampus during processing of structured tone sequences versus random tone sequences in 50% or more of participants. Prior studies have proposed that the hippocampus is highly relevant for SL, especially in the visual modality (Ellis et al., 2021; Schapiro et al., 2014, 2016, 2017; Turk-Browne et al., 2009). Specifically, a case study with a single patient with complete bilateral hippocampus loss and broader MTL damage revealed impaired SL in both linguistic and nonlinguistic domains and both visual and auditory modalities (Schapiro et al., 2014), indicating a causal role of the hippocampus in SL across domains and modalities. Similarly, Ramos-Escobar et al. (2022) identified activation of the hippocampus during auditory linguistic SL among seven pharmaco-resistant temporal lobe epilepsy patients. By examining event-related potentials, they demonstrated that the hippocampus was sensitive to word familiarity. However, the necessity of the hippocampus is still widely debated (Covington et al., 2018; Wang et al., 2023). A replication study of Schapiro et al. (2014) revealed that, in a larger group of patients with either localized hippocampus or broader MTL impairments, patients demonstrated above-chance performance in some SL tasks, although the patients still showed poorer learning outcomes compared to the unimpaired group (Covington et al., 2018). It is therefore proposed that SL can still occur in the absence of hippocampal activation. Furthermore, most studies advocating for the necessity of the hippocampus have focused on patient populations and visual SL (Ellis et al., 2021; Schapiro et al., 2014, 2017; Turk-Browne et al., 2009). Neurotypical populations may recruit different neural regions to support SL, and activation of the hippocampus may be domain and modality specific. Future studies should therefore directly compare the role of the hippocampus in neurotypical populations across a variety of modalities and domains.

### Limitations and Future Directions

Despite our findings regarding the domain-specific neural basis of SL, the current study was not without limitations. First, we hypothesize that the spatially segregated patterns of brain activation across linguistic and nonlinguistic domains is attributed to the ongoing maturation of children’s brain. However, without a comparison group, such as older children or adults, it is difficult to directly verify this hypothesis and we can only compare the current study with existing studies in adults with similar paradigms. Prior research has identified domain-general brain regions associated with SL in adults (Batterink et al., 2019), such as the left IFG, although most studies among adults have only focused on one domain (linguistic: McNealy et al., 2006; nonlinguistic: Abla & Okanoya, 2008). Based on existing research in adults, we expect that some domain-general regions, such as the left IFG, should show activation during both linguistic and nonlinguistic SL if the current paradigm was used with adults (Batterink et al., 2019). Future studies exploring the neural basis of SL should take both domains and different age groups into consideration.

Second, the presentation rate of stimuli in the present study may not be optimal for detecting behavioral differences in SL performance. While a slower rate of presentation was utilized to accurately measure changes in the BOLD signal (which was still faster than that recommended by Miezin et al., 2000), the presentation rate of stimuli in the current study was 2.1 Hz for both the syllable and tone condition. However, a recent behavioral study suggests that the optimal presentation rate for syllables and tones during SL is 4.5 Hz and 1.8 Hz, respectively (Boeve et al., 2024). Previous studies have also shown that slower presentation rates particularly harm auditory SL of syllable structures (e.g., Conway & Christiansen, 2009; Emberson et al., 2011). Presenting both syllable and tone regularities at their corresponding optimal rates may promote behavioral learning and maximize the engagement of brain regions. Additionally, the present study only compared SL of auditory linguistic and nonlinguistic regularities, but SL of linguistic and nonlinguistic regularities also occurs in other modalities such as the visual modality. Combining both auditory and visual modalities and using the optimal presentation rate for each stimulus type will provide a more comprehensive and accurate description of how domain and modality differentially modulate the neural basis of SL in children.

Third, the present study averages across the time course of SL. SL unfolds over time, as demonstrated by decreases in behavioral RT (Siegelman et al., 2018). This behavioral change is likely subserved by changes in how perceptual, executive, and language networks are activated across time to predict statistical regularities. Therefore, future research should consider examining the neural basis of SL across time.

Fourth, our behavioral results were based on a threshold of six or more valid responses based on previous research (Hu et al., 2024; Schneider et al., 2020). Although this threshold is relatively low, the implicit nature of SL suggests that learning should still be possible regardless of behavioral performance, which was verified by *A*′ values above chance levels across all participants. Despite *A*′ values above chance levels, this threshold did limit the number of participants with feasible behavioral and neuroimaging data, which resulted in relatively small effect sizes and low power. Furthermore, this lack of power may be the reason we failed to uncover significant differences in mean RT between structured and random sequences in the 0–960 ms window. The interpretation of the paired sample *t* tests should also be cautious considering the low sample size. While our findings provide preliminary evidence for a domain-specific mechanism supporting SL across domains in young children, future research should include additional participants to increase power and better inform our understanding of the neural mechanisms that underlie SL across domains.

Fifth, children’s brains are still developing and therefore, highly heterogeneous (Cui et al., 2020). In the current study we take an individual-subjects approach to limit the degree of heterogeneity present; however, we averaged across children ages 5–7 years. Given the developing brain is undergoing substantial functional and structural changes during this time, it is possible that there is greater conjunction across tasks within each age group. At the same time, the current heterogeneity in our behavioral findings may underlie distinct patterns of neural activation. Specifically, in the current study, a number of children did demonstrate behavioral learning of statistical regularities (i.e., shorter RT in the structured sequences than in the random sequences), while others did not. It could be the case that children who demonstrated behavioral learning had higher degrees of conjunction than those who did not show behavioral learning. Unfortunately, due to the aforementioned small sample size dictated by our behavioral data, we were unable to disentangle whether there were distinct neural profiles between children who did and did not demonstrate behavioral learning. Future studies should carefully consider approaches for disentangling heterogeneity associated with brain maturation and behavioral performance which may impact the neural patterns presented in the current study.

Despite these limitations, the current investigation into the neural basis of auditory SL across linguistic and nonlinguistic domains demonstrates that the functional organization of SL in the developing brain may rely on domain-specific mechanisms. The lack of conjunction between nonlinguistic and linguistic domains might be due to the immaturity and heterogeneity of developing children’s brains. This ongoing neural specialization and heterogeneity of children’s brains is perhaps critical for language acquisition though, because, as opposed to the mature brain, it allows for children to be more adaptable to their language learning environments. In sum, the present study directly explores and compares the neural basis of SL of nonlinguistic and linguistic auditory regularities and provides new evidence for a domain-specific neural basis of learning during the early developmental stage (Karmiloff-Smith, 2018).

## ACKNOWLEDGMENTS

We thank Dr. Zhenghan Qi for her original design of the experiment and mentorship during data collection, Dr. Terri Scott for the sharing of her neuroimaging analysis scripts, and Drs. Jason Scimeca, Christopher Cox, Tehila Nugiel, Andrew Lynn, and Eric Wilkey for their consultation on the neuroimaging analyses.

## FUNDING INFORMATION

Julie Schneider, SBE Office of Multidisciplinary Activities (https://dx.doi.org/10.13039/100005717), Award ID: 1911462. Julie Schneider, Developmental Sciences, Award ID: 2141007.

## AUTHOR CONTRIBUTIONS

**Tengwen Fan**: Formal analysis; Writing – original draft. **Will Decker**: Writing – review & editing. **Julie Schneider**: Conceptualization; Funding acquisition; Investigation; Methodology; Supervision; Writing – review & editing.

## Supplementary Material



## TECHNICAL TERMS

Statistical learning: The ability to extract and monitor regularities from the environment across time; often considered a fundamental mechanism of learning.
Domain-general: Relates to the idea that humans are born with mechanisms in the brain that exist to support and guide learning on a broad level, regardless of the type of information being learned.
Domain-specific: Refers to the idea that many aspects of cognition are supported by specialized, presumably evolutionarily specified, learning devices.
Watershed algorithm: Uses topographic information to divide an image into multiple segments or regions. The algorithm views an image as a topographic surface, each pixel representing a different magnitude.
