# Supplementary material for: The Domain-Specific Neural Basis of Auditory Statistical Learning in 5–7-Year-Old Children
Source: Neurobiol Lang (Camb). 2024 Oct 28;5(4):981–1007. doi: 10.1162/nol_a_00156 (PMC11527419; doi:10.1162/nol_a_00156)
Supplement: Supplementary file 1 [file nol-5-4-981-s001.pdf]

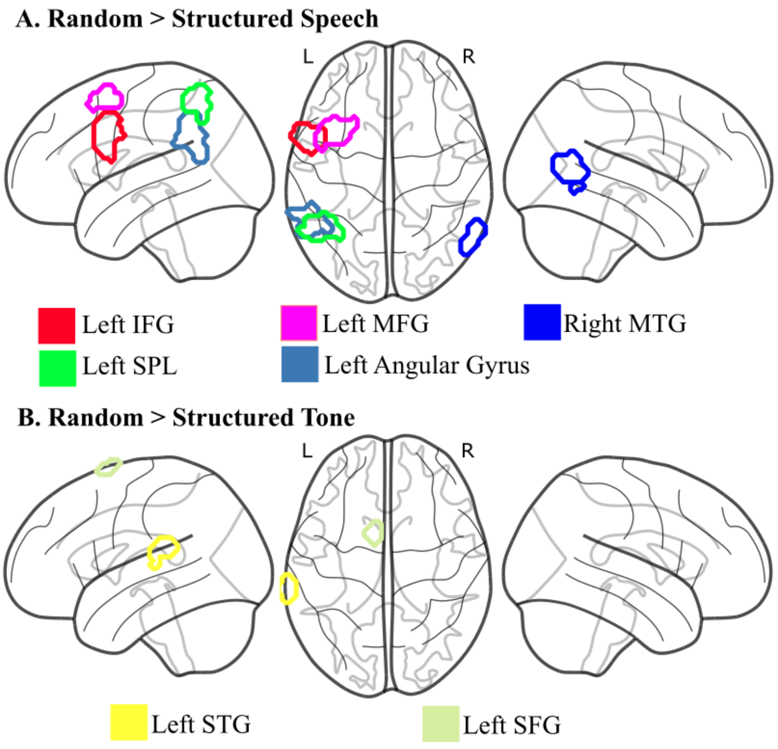

Supplementary Figure 1. GCSS analysis. Parcels representing significant activation ($z > 2.326$) for random > structured contrast in both syllable and tone tasks across more than 60% of participants. IFG: Inferior Frontal Gyrus; MFG: Middle Frontal Gyrus; SFG: Superior Frontal Gyrus; STG: Superior Temporal Gyrus; MTG: Middle Temporal Gyrus; SPL: Superior Parietal Lobule.

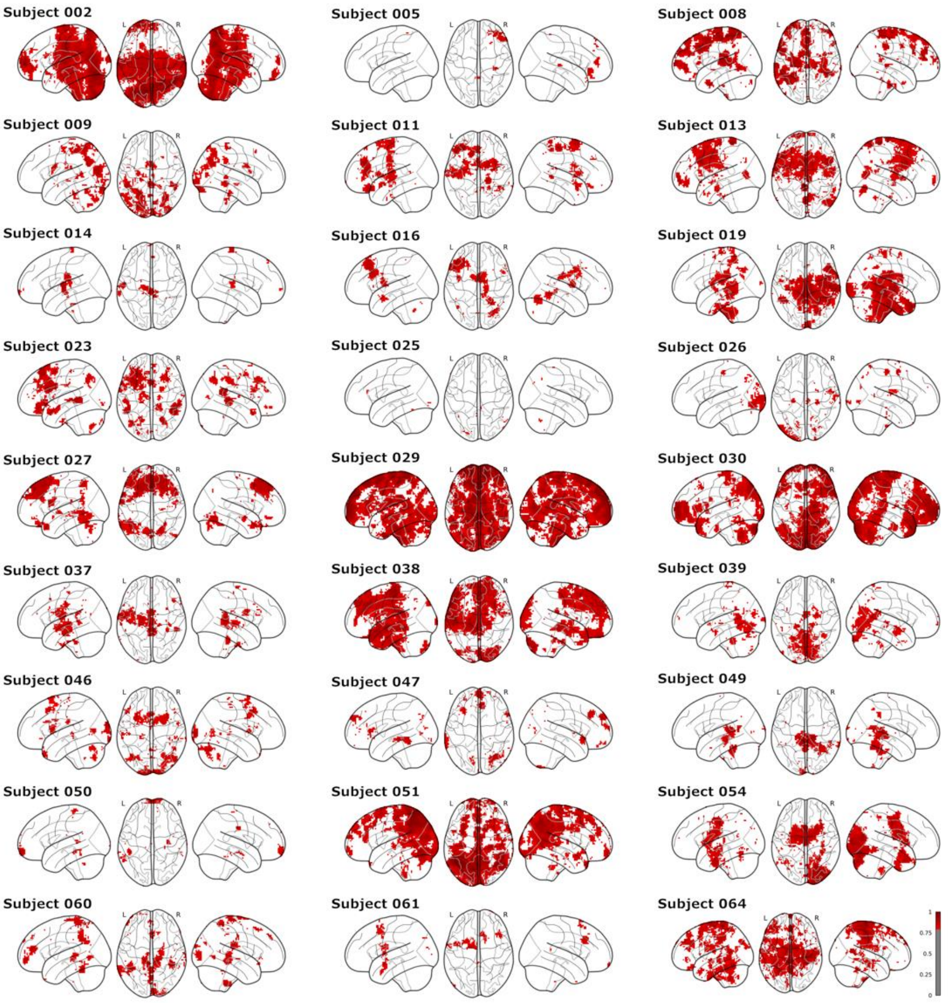

Supplementary Figure 2. Individual whole-brain correlations across linguistic and non-linguistic SL tasks. Correlation *r* values across tasks at each voxel, thresholded at .80, are plotted for each individual participant.

Supplementary Table 1. Number of behavioral responses per subject in the time window of 0-960ms. The total target number was 48 in both syllable and tone conditions.

Subject ID	Syllable Total Hits	Tone Total Hits	Inclusion
001	1	3	Removed
002	0	1	Removed
005	7	6	Kept
006	19	14	Kept
007	27	16	Kept
008	1	0	Removed
009	47	44	Kept
011	30	22	Kept
013	21	30	Kept
014	3	8	Kept for Tone
016	11	4	Kept for Syllable
019	0	0	Removed
023	45	46	Kept
025	19	20	Kept
026	25	0	Kept for Syllable
027	5	6	Kept for Tone
029	35	10	Kept
030	4	10	Kept for Tone
037	21	0	Kept for Syllable
038	38	35	Kept
039	31	0	Kept for Syllable
046	35	3	Kept for Syllable
047	0	0	Removed
049	10	0	Kept for Syllable
051	24	17	Kept
054	1	0	Removed
060	1	0	Removed
061	7	2	Kept for Syllable
064	15	7	Kept

Note: Subject 050 has neuroimaging data but no behavioral data. Eleven participants in the syllable condition and15 participants in the tone condition were removed from the RT analysis.

Supplementary Table 2. Paired t tests for the log-transformed RT between each structured and random block of the syllable task.

	M (*SD*) Syllable<br>Structured log RT	*M* (*SD*) Syllable<br>Random log RT	*t*	*p*
Block 1	5.97 (0.43)	5.84 (0.78)	$t\,(12) = 0.73$	$p = 0.48$
Block 2	5.95 (0.52)	5.92 (0.63)	$t\,(12) = 0.14$	$p = 0.89$
Block 3	6.01 (0.40)	6.01 (0.49)	$t\,(16) = -0.24$	$p = 0.81$

Supplementary Table 3. Paired t tests for the log-transformed RT between each structured and random block of the tone task.

	M (*SD*) Tone<br>Structured log RT	*M* (*SD*) Tone<br>Random log RT	*t*	*p*
Block 1	5.77 (0.45)	5.90 (0.32)	$t\,(6) = -1.24$	$p = 0.26$
Block 2	5.64 (0.61)	5.94 (0.43)	$t\,(9) = -1.25$	$p = 0.24$
Block 3	6.02 (0.50)	5.89 (0.41)	$t\,(8) = 0.79$	$p = 0.45$

Supplementary Table 4. Individual behavioral performance on the linguistic and non-linguistic SL tasks as measured by A’ values.

Participant	Linguistic SL_A’	Non-linguistic SL_A’
001	0.593	0.645
002	0.249	0.662
005	0.706	0.684
006	0.692	0.668
007	0.690	0.649
008	0.662	
009	0.768	0.700
011	0.875	0.682
013	0.550	0.588
014	0.597	0.733
016	0.626	0.648
023	0.751	0.757
025	0.767	0.680
026	0.770	0.250
027	0.676	0.509
029	0.691	0.676
030	0.746	0.714
037	0.835	0.250
038	0.687	0.681
039	0.891	
046	0.589	0.637
049	0.602	0.249
051	0.725	0.619
054	0.755	
060	0.639	
061	0.561	0.666
064	0.596	0.717

Supplementary Table 5. Paired t tests for *A*' between each structured and random block of the syllable task.

	M (*SD*) Syllable Structured A'	*M* (*SD*) Syllable Random A'	*t*	*p*
Block 1	0.72 (0.21)	0.61 (0.24)	*t* (17) = 1.80	*p* = 0.09
Block 2	0.67 (0.20)	0.68 (0.19)	*t* (15) = -0.16	*p* = 0.87
Block 3	0.71 (0.19)	0.71 (0.13)	*t* (17) = -0.04	*p* = 0.97

Supplementary Table 6. Paired t tests for *A*' between each structured and random block of the tone task.

	M (*SD*) Tone Structured A'	*M* (*SD*) Tone Random A'	*t*	*p*
Block 1	0.53 (0.25)	0.65 (0.21)	*t* (14) = -1.27	*p* = 0.22
Block 2	0.54 (0.23)	0.69 (0.15)	*t* (13) = -2.21	*p* = 0.05
Block 3	0.65 (0.16)	0.55 (0.23)	*t* (12) = 1.30	*p* = 0.22

Supplementary Table 7. Quality control: the Framewise Displacement (FD) values for each subject included in the neuroimaging analysis.

Participant	FD for Run 1	FD for Run 2	Mean FD across runs	Inclusion
001	2.053	2.783	2.418	Removed
002	0.689	0.699	0.694	Kept
005	0.734	0.934	0.834	Kept
006	2.798	1.689	2.2435	Removed
007	1.466	2.069	1.7675	Removed
008	0.172	0.178	0.175	Kept
009	0.217	0.209	0.213	Kept
011	0.459	0.576	0.5175	Kept
013	0.583	0.605	0.594	Kept
014	0.401	0.286	0.3435	Kept
016	0.896	0.782	0.841	Kept
019	0.242	0.515	0.3785	Kept
023	0.158	0.161	0.1595	Kept
025	0.445	1.133	0.789	Kept
026	0.144	0.155	0.1495	Kept
027	0.246	0.829	0.5375	Kept
029	0.197	0.243	0.22	Kept
030	0.549	1.121	0.835	Kept
037	0.182	0.281	0.2315	Kept
038	0.371	0.387	0.379	Kept
039	0.234	0.259	0.2465	Kept
046	1.498	1.52	1.509	Kept
047	0.388	0.255	0.3215	Kept
049	0.293	0.261	0.277	Kept
050	0.213	0.283	0.248	Kept
051	0.146	0.217	0.1815	Kept
054	0.309	0.277	0.293	Kept
060	0.738	0.836	0.787	Kept
061	1.007	0.769	0.888	Kept
064	0.16	0.181	0.1705	Kept
